# Interventions to reduce pharmacologic opioid exposure in neonatal opioid withdrawal syndrome: a systematic review and meta-analysis of randomized studies

**DOI:** 10.3389/fpain.2026.1826942

**Published:** 2026-04-29

**Authors:** Muhammad Ahmad, Ayoola Awosika, Chinenye Iguh, Wagma Khan, Neha Choudhary, Marya Aisha, Fatima Shaukat, Soura Rajeshwara, Maheen Zahid, Muneeba Shaukat

**Affiliations:** 1 Department of Pediatrics, King Edward Medical University, Lahore, Pakistan; 2 Department of Family Medicine, University of Illinois College of Medicine Peoria, Bloomington, IL, United States; 3 Department of Medicine, Windsor University School of Medicine, Cayon, Saint Kitts and Nevis; 4 Department of Pediatrics, District Headquarters Teaching Hospital Mardan, Mardan KPK, Pakistan; 5 Punjab Health and Population Department, Jubilee Hospital, Bahawalpur, Pakistan; 6 Department of Pediatrics, Ameeruddin Medical College, Lahore, Pakistan; 7 Department of Psychiatry, Shimoga Institute of Medical Sciences, Shimoga, India

**Keywords:** eat–sleep–console therapy, neonatal abstinence syndrome, neonatal opioid withdrawal syndrome, pediatric pain, pharmacologic opioid exposure

## Abstract

**Background:**

Neonatal opioid withdrawal syndrome (NOWS) is increasingly prevalent and is frequently managed with pharmacologic opioid therapy, raising concerns regarding early-life opioid exposure and prolonged hospitalization. Interventions aimed at reducing pharmacologic exposure while maintaining safety have emerged, most notably care-model–based approaches. This study aims to systematically review the evidence evaluating interventions designed to reduce pharmacologic opioid exposure in neonates with NOWS compared with standard care.

**Methods:**

A systematic review was conducted in accordance with PRISMA guidelines. PubMed/MEDLINE, Cochrane CENTRAL, and Google Scholar were searched from inception through the final search date. Randomized controlled trials, comparative observational studies, and quality-improvement studies evaluating exposure-reduction strategies were included. Interventions of interest primarily comprised care-model approaches such as Eat–Sleep–Console (ESC). Outcomes included duration of opioid therapy, cumulative opioid exposure, length of hospital stay, and safety outcomes. Due to substantial heterogeneity across studies, findings were synthesized narratively.

**Results:**

Six studies met inclusion criteria, comprising three randomized studies and three observational or quality-improvement studies. Meta-analysis of randomized evidence showed ESC-based care significantly reduced hospital stay length (MD −6.50 days; 95% CI −9.63 to −3.36; *p* < 0.0001; I^2^ = 0%) and decreased opioid therapy duration (MD −3.06 days; 95% CI −3.74 to −2.38; *p* < 0.00001; I^2^ = 36%) compared to standard care. A subgroup analysis showed lower cumulative opioid exposure (MD −4.1 MME/kg; *p* = 0.001). Observational and quality-improvement studies consistently reported substantial reductions in opioid exposure and hospitalization following ESC implementation. Across all included studies reporting safety outcomes, no increase in adverse events or hospital readmissions was observed.

**Conclusions:**

Care-model interventions emphasizing functional assessment and nonpharmacologic support—particularly the ESC approach—are associated with reduced pharmacologic opioid exposure and shorter hospitalization for infants with NOWS without compromising short-term safety. These findings support evolving guideline recommendations favoring exposure-reduction strategies in NOWS management.

## Introduction

Neonatal opioid withdrawal syndrome (NOWS), also referred to as neonatal abstinence syndrome (NAS), is a growing public health concern that has paralleled the global opioid epidemic (1). Increasing rates of prenatal opioid exposure have led to a corresponding rise in the incidence of NOWS, resulting in substantial morbidity among affected neonates and significant strain on healthcare systems. Infants with NOWS frequently require prolonged hospitalization, specialized neonatal care, and pharmacologic treatment to manage withdrawal symptoms, contributing to increased healthcare utilization and costs (2, 3).

Pharmacologic therapy remains the cornerstone of treatment for moderate to severe NOWS, most commonly involving opioid replacement therapy followed by gradual dose tapering (4). While effective in controlling withdrawal symptoms, conventional weaning protocols are often associated with extended durations of opioid exposure and prolonged length of hospital stay (5). Notably, there is considerable variability in weaning practices across institutions, reflecting the absence of standardized, evidence-based approaches (6). Prolonged opioid exposure during a critical period of neurodevelopment has raised concerns regarding potential short- and long-term adverse outcomes (7), underscoring the need for strategies that minimize pharmacologic treatment while maintaining clinical safety.

In response to these concerns, a range of interventions aimed at reducing pharmacologic opioid exposure have emerged. These include accelerated or shortened opioid weaning protocols (8), standardized treatment pathways, and care-model interventions that emphasize functional assessment and non-pharmacologic support. Approaches such as protocol-driven care and Eat–Sleep–Console (ESC)–based models have been associated with reduced need for pharmacologic treatment (9), shorter duration of opioid therapy, and decreased length of hospital stay in some settings (10). The Eat–Sleep–Console (ESC)–based care approach focuses on functional assessment rather than scoring systems: the infant is evaluated on ability to eat adequately, sleep ≥1 h, and be consoled within ∼10 min. Management prioritizes non-pharmacologic interventions (rooming-in, breastfeeding, skin-to-skin), with medications used only if these goals are not met. Additionally, selected programs have explored earlier transition to outpatient weaning as a means of reducing inpatient opioid exposure (10). Despite promising results, these interventions vary widely in design, implementation, and reported outcomes (11).

Although several systematic reviews and meta-analyses have examined pharmacologic agents used in the treatment of NOWS, existing syntheses have largely focused on comparisons between opioid medications rather than strategies intended to reduce overall opioid exposure (12). Consequently, the effectiveness and safety of exposure-reducing interventions—particularly accelerated weaning and protocolized care approaches—have not been comprehensively evaluated. This gap in the literature contributes to ongoing clinical uncertainty and heterogeneity in practice.

Therefore, the aim of this systematic review is to evaluate the effectiveness of interventions designed to reduce pharmacologic opioid exposure in neonates with neonatal opioid withdrawal syndrome compared with standard care. We synthesize evidence on their impact on duration of opioid therapy, length of hospital stay, cumulative opioid exposure, and treatment-related outcomes. By integrating findings from randomized and comparative observational studies, this review aims to inform clinical decision-making and support the development of evidence-based strategies to optimize care for infants with NOWS.

## Materials and methods

This study was conducted in accordance with the Preferred Reporting Items for Systematic Reviews and Meta-Analyses (PRISMA) 2020 guidelines (13). A comprehensive literature search was conducted in PubMed, Cochrane Central Register of Controlled Trials (CENTRAL), and Google Scholar from database inception to the date of final search. Search strategies included keywords such as “Neonatal Abstinence Syndrome,” Neonatal Opioid Withdrawal Syndrome, “opioid,” “ morphine,” “methadone,” “buprenorphine,” “wean,” and “Eat Sleep Console”. Database-specific search strings were developed to optimize sensitivity and are provided in the supplementary material. Reference lists of included studies and relevant reviews were manually screened to identify additional eligible studies.

### Eligibility criteria

Eligible studies included neonates diagnosed with NOWS or NAS following in-utero opioid exposure. Interventions of interest were those explicitly designed to reduce pharmacologic opioid exposure, including accelerated or shortened opioid weaning protocols, protocol-driven dose reduction strategies, care-model interventions such as ESC–based approaches, and early transition to outpatient weaning when intended to reduce inpatient opioid exposure. Comparators consisted of standard or usual opioid weaning protocols, conventional pharmacologic management, or pre-intervention usual-care cohorts.

Studies were required to report at least one prespecified outcome of interest, including duration of pharmacologic opioid therapy, length of hospital stay, cumulative opioid dose, treatment escalation, or adverse events. Randomized controlled trials (individual or cluster), quasi-randomized trials, and comparative observational studies (prospective or retrospective) were eligible for inclusion. Case reports, case series without a comparator, editorials, commentaries, narrative reviews, and studies comparing opioid agents without differences in exposure duration or weaning strategy were excluded.

### Study selection

All retrieved records were imported into reference management software, and duplicate citations were removed. Two reviewers independently screened titles and abstracts to identify potentially eligible studies. Full-text articles of selected records were then independently assessed for eligibility using the predefined inclusion and exclusion criteria. Discrepancies at any stage of the screening process were resolved through discussion, and when necessary, consultation with a third reviewer. The study selection process was documented using a PRISMA flow diagram.

### Data extraction

Data extraction was performed independently by two reviewers using a standardized, pilot-tested data extraction form to ensure consistency and reproducibility. Extracted data included study characteristics (author, year, country, study design), population characteristics (sample size, gestational age, type of prenatal opioid exposure), intervention and comparator details (type of intervention, assessment or weaning strategy, duration), and reported outcomes.

For continuous outcomes, means and standard deviations were extracted where available. When studies reported medians and interquartile ranges, these were recorded as reported; no statistical conversions were undertaken for the purpose of quantitative pooling. Dichotomous outcomes were extracted as event counts and total sample sizes. Any discrepancies in extracted data were resolved by consensus between reviewers.

### Outcomes

The primary outcome of interest was the total duration of pharmacologic opioid therapy, defined as the number of days from initiation to cessation of opioid treatment for neonatal opioid withdrawal syndrome. Secondary outcomes included length of hospital stay, cumulative opioid exposure, need for treatment escalation or adjunctive therapy, and adverse events, including seizures, respiratory depression, or hospital readmission. Where reported, feeding-related outcomes such as breastfeeding at discharge were also extracted.

### Risk of bias assessment

Risk of bias was assessed independently by two reviewers. Randomized controlled trials were evaluated using the Cochrane Risk of Bias 2.0 tool (14), while non-randomized studies were assessed using the ROBINS-I tool (15). Each study was categorized as having low, moderate, serious, or critical risk of bias. Disagreements were resolved through discussion or, when necessary, adjudication by a third reviewer.

### Data synthesis

Given anticipated clinical and methodological heterogeneity across included studies—including differences in intervention type, study design, outcome definitions—a mixed synthesis approach was adopted. RCTs with comparable outcome measures were quantitatively synthesized using Revman 5.4.1. Continuous outcomes, including length of hospital stay and duration of opioid therapy, were pooled using a fixed-effects model with inverse-variance weighting, and results were reported as mean differences (MD) with 95% confidence intervals (CIs). Where standard deviations (SD) were not reported, they were estimated from available CIs using established methods.

Non-randomized studies, including observational and quality-improvement (QI) designs, were not pooled quantitatively due to lack of comparable effect measures, absence of variance data, and inherent methodological limitations. These studies were instead synthesized narratively. Findings were grouped according to intervention type (care-model interventions such as ESC, accelerated pharmacologic weaning strategies, and other exposure-reduction approaches) and study design. The direction, magnitude, and consistency of effects on primary and secondary outcomes were described.

### Subgroup and sensitivity considerations

Subgroup analyses were performed qualitatively based on study design (randomized vs. non-randomized) and intervention type (ESC-based care vs. pharmacologic optimization strategies) to explore consistency of findings across different clinical contexts. Quantitative subgroup analyses were not conducted due to the limited number of studies within each category.

Sensitivity analyses were not formally performed; however, the robustness of findings was assessed by considering study design, sample size, and risk of bias. Greater weight was given to evidence derived from randomized trials, while findings from observational and QI studies were interpreted cautiously in light of their higher risk of bias and potential confounding.

## Results

**Figure 1:**
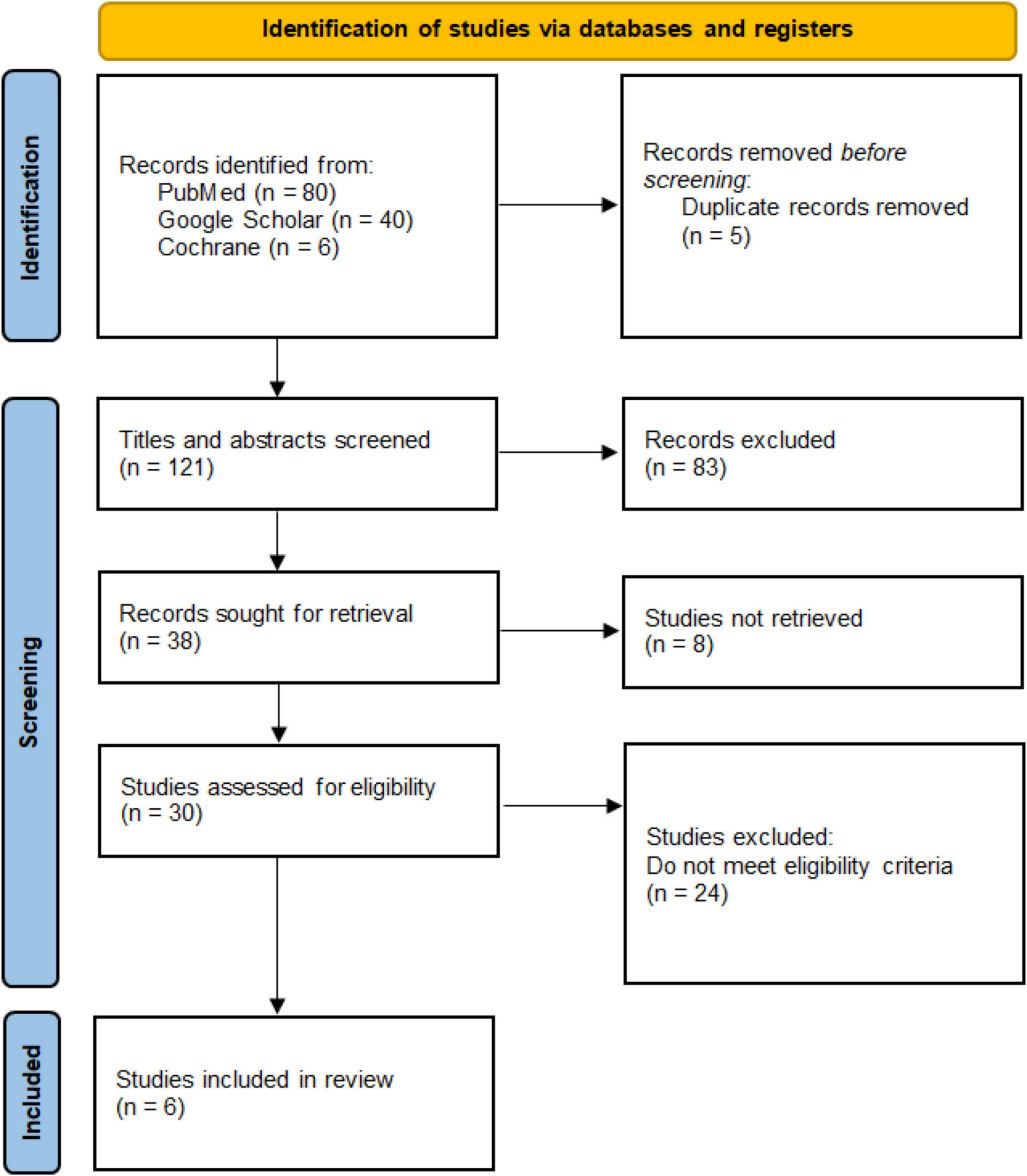
PRISMA flowchart.

The database search identified a total of 126 records. After removal of duplicates, 121 records underwent title and abstract screening, of which 83 were excluded for not meeting inclusion criteria. Full-text review was performed for 30 articles, resulting in six studies meeting eligibility criteria for inclusion in the systematic review (Figure 1). These comprised three completed randomized controlled trials and three observational or quality-improvement studies.

### Study characteristics

The characteristics of included studies are summarized in Table 1. All studies were conducted in the United States and published between 2017 and 2026. Study designs included a multicenter stepped-wedge cluster randomized controlled trial, a *post hoc* subgroup analysis of a randomized trial, one multicenter randomized trial, one retrospective observational study, and two quality-improvement before–after studies.

**Table 1:** Characteristics of included studies.

Study	Year	Country	Study Design	Setting	Population (N)	Intervention	Comparator	Key Outcomes Reported
Young et al. ( 16 )	2023	USA	Stepped-wedge cluster RCT	Multicenter neonatal units (26 hospitals)	1,305 infants ≥36 weeks	ESC care model	Usual care (FNASS-based)	Time to medical readiness, length of stay, receipt of opioid therapy, safety
Devlin et al. ( 17 )	2024	USA	Post hoc subgroup analysis of cluster RCT	Multicenter neonatal units	463 pharmacologically treated infants	ESC care model	Usual care (FNASS-based)	Duration of opioid therapy, cumulative opioid exposure, length of stay, safety
Laptook et al. ( 18 )	2026	USA	Multicenter RCT	Multiple neonatal centers	189 infants ≥36 weeks	Accelerated opioid weaning (15% dose decrements)	Standard weaning (10% decrements)	Duration of opioid therapy, safety outcomes
Grossman et al. (19)	2017	USA	Quality-improvement before–after study	Single-center inpatient unit	287 infants ≥35 weeks	ESC-based care with PRN morphine and standardized nonpharmacologic care	Pre-implementation usual care	Length of stay, morphine exposure, NICU utilization, costs
Grossman et al. (20)	2018	USA	Retrospective observational study	Single-center inpatient unit	50 infants ≥35 weeks	ESC assessment approach	FNASS-predicted management	Morphine initiation/escalation, length of stay, safety
Blount et al. ( 21 )	2019	USA	Quality-improvement before–after study	Single-center inpatient floor	76 infants ≥35 weeks	ESC-based care with PRN morphine and reinforced nonpharmacologic care	Baseline FNASS-based care	Length of stay, opioid exposure, adverse events

ESC, eat, sleep, console; FNASS, Finnegan Neonatal Abstinence Scoring System; RCT, randomized controlled trial; NICU, Neonatal Intensive Care Unit; QI, quality improvement.

The included population consisted of neonates with confirmed prenatal opioid exposure diagnosed with NOWS, predominantly term or near-term infants (≥35–36 weeks’ gestation). Interventions were broadly categorized as care-model interventions—primarily the ESC approach—or pharmacologic exposure–reduction strategies, including accelerated opioid weaning protocols and symptom-based (as-needed) opioid dosing. Comparators consisted of usual care practices, most commonly based on the Finnegan Neonatal Abstinence Scoring System (FNASS) with scheduled opioid dosing and tapering.

### Effects of care-model interventions (ESC)

#### Randomized evidence

**Figure 2:**
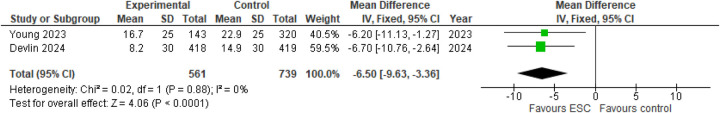
Forest plot comparing the effect of the ESC care model vs. usual care on length of hospital stay in neonates with NOWS. Studies included are: (16, 17).

Two studies provided high-quality randomized evidence evaluating the ESC care model. In a large multicenter stepped-wedge cluster randomized trial involving 26 hospitals, implementation of ESC was associated with a substantial reduction in time to medical readiness for discharge compared with usual FNASS-based care. A meta-analysis of two randomized studies demonstrated that the ESC care model significantly reduced length of hospital stay compared with usual care. Individually Young et al. (16) reported a mean reduction of 6.7 days (95% CI: −10.76 to −2.64), while Devlin et al. (17) reported a mean reduction of 6.2 days (95% CI: −11.13 to −1.27). Pooled analysis using a fixed-effects model showed mean difference: −6.50 days (95% CI: −9.63 to −3.36; *p* < 0.0001). There was no observed heterogeneity between studies [I^2^ = 0%, *χ*^2^ = 0.02 (*p* = 0.88)] (Figure 2).

**Figure 3:**
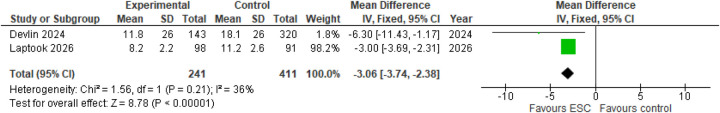
Forest plot comparing exposure-reduction strategies (including ESC-based care and accelerated weaning protocols) vs. standard care on duration of pharmacologic opioid therapy in neonates with NOWS. Studies included are: (17, 18).

A meta-analysis of two randomized studies demonstrated that exposure-reduction strategies significantly decreased the duration of pharmacologic opioid therapy compared with standard care. Individually Devlin et al. (17) reported a mean reduction of 6.3 days (95% CI: −11.43 to −1.17) and Laptook et al. (18) reported a mean reduction of 3.0 days (95% CI: −3.69 to −2.31). Pooled analysis using a fixed-effects model showed mean difference: −3.06 days (95% CI: −3.74 to −2.38; *p* < 0.00001). There was low-to-moderate heterogeneity between studies [I^2^ = 36%, *χ*^2^ = 1.56 (*p* = 0.21)] (Figure 3).

Additionally, ESC-managed infants had a shorter overall hospital length of stay, with no increase in peak opioid dose, need for adjunctive medications, or serious adverse events.

### Observational and quality-improvement evidence

Three single-center studies provided observational and quality-improvement evidence supporting the effectiveness of ESC-based interventions. Across these studies, implementation of ESC—often combined with reinforced nonpharmacologic care and as-needed opioid dosing—was consistently associated with large reductions in length of stay and pharmacologic opioid exposure.

In a large quality-improvement initiative spanning multiple Plan–Do–Study–Act cycles (19), average length of stay decreased by more than 70%, and the proportion of infants treated with morphine declined dramatically following adoption of ESC-based care and functional assessment strategies. Similarly, a retrospective observational study comparing actual ESC-guided treatment decisions vs. FNASS-predicted management found that the ESC approach substantially reduced morphine initiation and escalation without reported adverse events.

Another quality-improvement study conducted on an inpatient pediatric floor demonstrated that transitioning from FNASS-based assessment to ESC, coupled with use of as-needed morphine and rapid tapering when required, resulted in a greater than 50% reduction in length of stay and near elimination of scheduled opioid therapy (21). No increase in NAS-related readmissions or serious adverse events was observed.

### Accelerated pharmacologic weaning strategies

One multicenter randomized clinical trial evaluated an accelerated opioid weaning protocol among infants receiving morphine or methadone for NOWS. Infants randomized to a 15% dose-decrement weaning strategy experienced a significantly shorter duration of opioid therapy compared with those receiving a standard 10% decrement protocol (18). The accelerated weaning approach was well tolerated, with few adverse events reported and no significant safety concerns identified.

### Safety outcomes

Across all completed studies reporting safety outcomes, interventions designed to reduce pharmacologic opioid exposure were not associated with an increased risk of adverse events. Randomized trial data demonstrated no difference in serious adverse outcomes between ESC and usual care groups. Observational and quality-improvement studies similarly reported no increase in seizures, respiratory complications, or NAS-related readmissions following implementation of ESC-based care or opioid exposure–reduction strategies (Table 2).

**Table 2:** Summary of interventions and Key outcomes.

Study	Intervention Category	Description of Intervention	Length of Stay	Opioid Therapy Duration / Exposure	Safety Outcomes
Young et al. (16)	Care-model intervention (ESC)	Functional assessment emphasizing eating, sleeping, consolability with optimized nonpharmacologic care	↓	↓ proportion receiving opioids	No increase
Devlin et al. (17)	Care-model intervention (ESC)	ESC applied to pharmacologically treated infants	↓	↓ duration and cumulative opioid dose	No increase
Laptook et al. (18)	Pharmacologic optimization	Accelerated opioid weaning with 15% dose decrements	—	↓ duration of opioid therapy	No increase
Grossman et al. (19)	Care-model / QI	ESC-based care, PRN morphine, avoidance of NICU	↓↓↓	↓↓↓ opioid exposure	No adverse events
Grossman et al. (20)	Assessment-based	ESC assessment vs FNASS-predicted care	↓	↓ morphine initiation/escalation	No adverse events
Blount et al. (21)	Care-model / QI	ESC assessment with PRN morphine	↓↓	↓↓↓ opioid doses/exposure	No readmissions

ESC, ESC; FNASS, Finnegan Neonatal Abstinence Scoring System; QI, quality improvement; PRN, as needed; ↓ indicates reduction compared with comparator; ↓↓ moderate reduction; ↓↓↓ large reduction.

### Risk of bias within studies

Randomized controlled trials were assessed as having low risk of bias, with appropriate adjustment for clustering and temporal trends. The *post hoc* subgroup analysis was considered methodologically robust but subject to limitations inherent to secondary analyses. Observational and quality-improvement studies were judged to have serious risk of bias, primarily due to before–after designs, lack of concurrent control groups, and multicomponent interventions. These studies were therefore synthesized narratively rather than quantitatively.

## Discussion

This systematic review synthesizes contemporary evidence on interventions designed to reduce pharmacologic opioid exposure in neonates with NOWS. Across randomized trials, observational studies, and quality-improvement initiatives, interventions emphasizing functional assessment, nonpharmacologic care, and judicious opioid use—most notably the ESC care model—were consistently associated with reductions in duration of opioid therapy, cumulative opioid exposure, and length of hospital stay, without evidence of increased short-term adverse outcomes. Collectively, these findings support a paradigm shift in NOWS management away from symptom-score–driven escalation toward function-based, family-centered care that prioritizes minimizing pharmacologic exposure (22).

The most robust evidence identified in this review supports the ESC care model as an effective strategy for reducing opioid exposure in infants with NOWS. In pooled analysis of randomized trials, ESC-based care was associated with clinically meaningful reductions in length of hospital stay (approximately 6–7 days) and duration of opioid therapy (approximately 3–6 days) compared with standard care. These findings are consistent with individual studies demonstrating reductions in the proportion of infants requiring pharmacologic treatment and overall treatment burden (23–25). Importantly, these benefits were achieved without increases in peak opioid dosing, need for adjunctive therapy (26), or short-term safety events (27). These findings align with growing clinical adoption of ESC across diverse care settings and provide high-quality evidence to support its broader implementation (28).

Observational and quality-improvement studies further reinforce the real-world effectiveness of ESC-based interventions. Across these studies, implementation of ESC was associated with large absolute reductions in length of stay ranging from approximately 5 to 16 days, as well as marked decreases in opioid utilization, including substantial reductions in morphine exposure and treatment rates. Despite inherent methodological limitations, the consistency in both direction and magnitude of effect across diverse clinical settings and study designs strengthens confidence in the clinical relevance and generalizability of these findings. These results suggest that the benefits of ESC are not confined to controlled trial environments but are reproducible in routine clinical practice (29, 30).

Current clinical guidance increasingly emphasizes nonpharmacologic interventions as first-line therapy for NOWS (22) and encourages minimizing opioid exposure when pharmacologic treatment is required. The findings of this review provide empirical support for these recommendations and extend them by demonstrating that structured care-model interventions such as ESC can safely operationalize these principles at scale. As professional societies and health systems continue to update clinical pathways for NOWS, the evidence summarized here supports incorporating ESC-based assessment and care as a standard approach, rather than an adjunct or optional strategy.

Across all included studies reporting safety outcomes, interventions aimed at reducing pharmacologic opioid exposure were not associated with increased risks of seizures, respiratory compromise, or hospital readmission. While most studies focused on short-term outcomes, the absence of observed safety signals is reassuring and supports the clinical feasibility of these strategies. Nonetheless, continued monitoring of long-term neurodevelopmental and family-centered outcomes remains essential, particularly as exposure-reducing approaches become more widely implemented.

This review has several strengths, including a comprehensive search strategy, inclusion of high-quality randomized evidence, and a structured narrative synthesis that appropriately accounts for heterogeneity in study designs and interventions. By focusing specifically on pharmacologic exposure reduction rather than length of stay alone, this review addresses a clinically and biologically relevant outcome with important implications for infant development. The absence of statistical heterogeneity in length-of-stay outcomes (I^2^ = 0%) suggests highly consistent effects of ESC across randomized settings, whereas moderate heterogeneity observed for opioid duration (I^2^ = 36%) likely reflects differences in intervention implementation and pharmacologic protocols.

However, several limitations warrant consideration. First, although quantitative meta-analysis was feasible for randomized studies, substantial heterogeneity in study design, intervention components, and outcome reporting precluded pooled analysis across all included studies. While narrative synthesis is appropriate in this context, it limits the ability to estimate pooled effect sizes. Second, meta-analysis was limited to a small number of randomized studies, and some variance estimates were derived from reported confidence intervals, which may introduce imprecision in pooled estimates. Third, many ESC studies employed multicomponent interventions, making it difficult to isolate the independent effects of individual elements such as assessment strategy vs. pharmacologic dosing approach. Fourth, observational and quality-improvement studies were subject to serious risk of bias, including lack of concurrent controls and potential secular trends. Finally, long-term outcomes beyond early infancy were not consistently reported and remain an important evidence gap.

Future research should prioritize randomized evaluations of pharmacologic strategies that complement ESC-based care, including accelerated weaning protocols and symptom-based dosing approaches. Longitudinal studies examining neurodevelopmental, behavioral, and family-centered outcomes are also needed to fully characterize the long-term impact of reduced opioid exposure. Additionally, implementation research focused on scalability, equity, and caregiver engagement will be critical to ensure that exposure-reducing strategies benefit diverse populations and care settings.

## Conclusions

This review demonstrates that interventions designed to reduce pharmacologic opioid exposure—particularly the ESC care model—are associated with significant and clinically meaningful reductions in both hospital length of stay (approximately 6–7 days) and duration of opioid therapy (approximately 3–6 days) ininfants with NOWS, without compromising short-term safety. These findings provide robust quantitative support for current guideline trends emphasizing nonpharmacologic, function-based care and reinforce the ESC approach as a high-impact, evidence-based strategy for optimizing NOWS management. As ongoing trials further refine pharmacologic management strategies, integrating exposure-reduction principles into standard of care has the potential to improve outcomes for infants and families affected by prenatal opioid exposure.

## Data Availability

The original contributions presented in the study are included in the article/Supplementary material, further inquiries can be directed to the corresponding author.
